# Emergence of *Escherichia coli* producing OXA-48 β-lactamase in the community in Switzerland

**DOI:** 10.1186/s13756-015-0051-x

**Published:** 2015-03-26

**Authors:** Katrin Zurfluh, Magdalena T Nüesch-Inderbinen, Laurent Poirel, Patrice Nordmann, Herbert Hächler, Roger Stephan

**Affiliations:** Institute for Food Safety and Hygiene, Vetsuisse Faculty University of Zurich, Winterthurerstrasse 272, 8057 Zurich, Switzerland; Medical and Molecular Microbiology Unit “Emerging Antibiotic Resistance”, Dept of Medicine, Faculty of Science, University of Fribourg, Fribourg, Switzerland

**Keywords:** Carbapenemase, Community, Enterobacteriaceae, Dissemination, OXA-48

## Abstract

**Background:**

The emergence and worldwide spread of carbapenemase-producing Enterobacteriaceae is of great concern to public health services. The aim of this study was to investigate the occurrence of carbapenemase-producing Enterobacteriaceae in the community in Switzerland.

**Findings:**

One thousand and eighty-six stool samples of healthy humans (staff members of a food-processing company which were screened for the occurrence of salmonellae) were collected in September 2014. After an initial enrichment-step, carbapenemase-producing Enterobacteriaceae were isolated from the carbapenem-containing selective medium SUPERCARBA II. Grown colonies from 11 samples were screened by PCR for the presence of *bla*_KPC_, *bla*_NDM_, *bla*_OXA-48_ and *bla*_VIM_. A single OXA-48-producing *Escherichia coli* was detected. Phylogenetic grouping and multi-locus sequence typing (MLST) revealed that this strain belonged to D:ST38, a type which had been previously reported in the UK, France, Lebanon and Egypt.

**Conclusions:**

The results of this study show that OXA-48-producing Enterobacteriaceae have started to spread into the community in Switzerland, and a continuous monitoring is necessary to better understand their dissemination in the human population as well as in animals and the environment.

## Findings

Most carbapenemases identified in Enterobacteriaceae hydrolyze almost all β-lactam antibiotics, including carbapenems, with the exception of OXA-48 that weakly hydrolyses carbapenems but paradoxally spares broad-spectrum cephalosporins. Currently, the most prevalent carbapenemases found among Enterobacteriaceae worldwide include the Ambler class A carbapenemase KPC, the class B metallo-β-lactamases (MBLs) of the IMP-, NDM- or VIM-type, and the class D OXA-48-like oxacillinases [1].

Carbapenemase-producing Enterobacteriaceae (mainly *Klebsiella pneumoniae* and *Escherichia coli)* were first described in Europe in the 1990s, and since then they have been increasingly reported especially in clinical settings. The emergence of these bacteria is of great concern to public health services because they are often also co-resistant to many other antibiotic classes and thus, only few treatment options remain [2]. Their dissemination into the community and the environment is only a matter of time.

Two years ago, we reported that there was no evidence for the dissemination of carbapenemase-producing Enterobacteriaceae in the community in Switzerland [3]. The aim of this study was to survey the current situation for the presence of carbapenemase-producing Enterobacteriaceae in healthy humans in Switzerland.

In the context of a yearly routine fecal screening for salmonellae, stool samples were obtained from 3200 employees of a meat-processing company. This company consists of eight countrywide processing plants and employs people from the surrounding urban areas. Each staff member was tested only once. For this study, 1086 samples were randomly selected. The samples were tested for the presence of KPC-, NDM-, OXA-48, or VIM-type carbapenemase-producing Enterobacteriaceae. A loopful of each sample was enriched for 24 hours at 37°C in 5 mL of Enterobacteriaceae Enrichment (EE) broth without antibiotic (Oxoid, Ltd, Basingstoke, Hampshire, England). The enrichment culture was thereafter streaked onto a modified SUPERCARBA agar medium selecting for bacteria with reduced susceptibility to ertapenem, which is the most sensitive marker for carbapenemase production. The SUPERCARBA II medium differs from the previously published SUPERCARBA [4] since its basis is made of Trypticase Soy agar (rather than Drigalski) and contains vancomycin (10 μg/ml) and amphotericin B (5 μg/ml) in order to prevent the growth of Gram positives or fungi. SUPERCARBA, ideally complemented by the RAPIDEC® CARBA NP test (bioMérieux Ltd), has been shown to be most suited to detect carbapenemase producers, since it exhibits a very high specificity and a very high sensitivity even for those OXA-48-producing strains that often exhibit low MICs of carbapenems [4].

Growth of oxidase negative colonies was obtained for eleven selective plates and those colonies were screened by PCR for the presence of *bla*_KPC_, *bla*_NDM_, *bla*_OXA-48_ and *bla*_VIM_ using primers described previously [5,6]. Species identification using API ID 32 E (bioMérieux, Marcy l’Etoile, France) and multi-locus sequence typing [7] was performed with strains positive for any of the above listed genes. Phylogenetic classification was performed as described previously [8]*.*

A single isolate (JU-S-791) was found positive for the *bla*_OXA-48_ carbapenemase gene (100% identity after sequencing), which corresponded to a shedding prevalence of carbapenemase-producing Enterobacteriaceae in the tested collective of 0.09%. The Oxa-48-positive individual, a Turkish citizen born and raised in Switzerland, had no history of hospital admissions and no history of recent antibiotic therapy but reported annual visits to Turkey and a journey to Italy one month prior to sampling. The OXA-48 producer was identified as *E. coli* sequence type (ST) 38 and belonged to the extraintestinal pathogenic phylogenetic group D.

Susceptibility testing was performed by the agar diffusion method, using antibiotic disks (Becton Dickinson and Company, Maryland, USA) according to the manufacturers' protocols. Minimal inhibitory concentrations (MIC) of imipenem and cefotaxime, ceftazidime, and cefepime alone and in combination with clavulanic acid were determined by Etest strips (bioMérieux, Marcy l’Etoile, France). Results were interpreted according to the criteria of the Clinical and Laboratory Standards Institute (CLSI) [9].

The MIC of imipenem was 0.5 μg/ml, which is still below the susceptibility breakpoint defined by CLSI. Class D carbapenem-hydrolyzing β-lactamases possess weak carbapenemase-activity and usually do not confer a high-level resistance phenotype unless the strain exhibits additional permeability defects [10,11].

Although OXA-48-producers only very poorly hydrolyze expanded-spectrum cephalosporins, the strain JU-S-791 displayed an extended-spectrum β-lactamase (ESBL) phenotype (MIC of cefotaxime >16 μg/ml, and MIC of cefotaxime plus clavulanic acid = 0.5 μg/ml) and additionally tested resistant in the disk diffusion test to gentamicin (zone diameter 10 mm), nalidixic acid, (6.5 mm), sulfamethoxazole (6 mm) and trimethoprim (6 mm). It was susceptible in the disk diffusion test to ciprofloxacin (27 mm), tetracycline (19 mm), chloramphenicol (25 mm) and kanamycin (19 mm). Screening for *bla*_CTX-M_, *bla*_SHV_ and *bla*_TEM_ as described previously [12], revealed that the strain, in addition to the *bla*_OXA-48_ gene, co-harbored the ESBL gene *bla*_CTX-M-24_ and the narrow-spectrum β-lactamase gene *bla*_TEM-1_. In order to evaluate whether the *bla*_OXA-48_ gene could be transferred, conjugation experiments were performed using a standardized method for liquid mating techniques, as described elsewhere [13]. However, they systematically failed, suggesting a chromosomal location of this gene, and no further attempts at plasmid detection were undertaken.

OXA-48 was first described 2001 in a *K. pneumoniae* isolate from a patient in Istanbul, Turkey. It is suggested that the main reservoirs of OXA-48-harbouring *K. pneumoniae* and *E. coli* are in North African countries and Turkey [11]. Nowadays, OXA-48 producing Enterobacteriaceae are widely disseminated throughout many European countries and have led to several hospital outbreaks [11]. In particular, the spread of the *bla*_OXA-48_ gene in community isolates but also in the environment has been demonstrated in Morocco [14,15]. The successful spread of *bla*_OXA-48_ is linked to a single self-conjugative 62 kb IncL/M plasmid which was so far only identified in Enterobacteriaceae [16]. More recently, a chromosomal location of *bla*_OXA-48_ was reported from isolates in the UK, France, Egypt, Lebanon and Switzerland [17-19], not only among humans but also in fowl [20]. Interestingly, chromosomal location of *bla*_OXA-48_ was mostly associated with *E. coli* D:ST38 co-harboring *bla*_CTX-M-24_ and *bla*_TEM-1_—the same strain type described in this study. Considering these similarities, it is highly probable that the strain described in this study also contains a chromosomally encoded *bla*_OXA-48_.

In conclusion, our study demonstrates that the prevalence of carbapenemase-producing Enterobacteriaceae in healthy humans in Switzerland is 0.09% (95% CI = <0.0001-0.0057) and is associated with *E. coli* D:ST38. This particular strain has previously been predominantly associated with clinical settings [18,19,21]. Our results strongly suggest that this epidemic clone, harboring a non-transmissible *bla*_OXA-48_ gene, is currently circulating among healthy humans. Notably, this further demonstrates that the problem of the dissemination of *bla*_OXA-48_ positive isolates is not only related to the epidemicity of the widely identified plasmid pOXA-48a [22] among different strain backgrounds, but includes the dissemination of a single *E. coli* ST38 strain harboring the *bla*_OXA-48_ gene on its chromosome. It is now even more important that further studies are carried out in order to survey the dissemination of carbapenemase-producing enterobacterial strains in the population. As a strategy for ensuring the detection of strains that exhibit low MICs of carbapenems such as OXA-48-producers, the use of the SUPERCARBA II may be considered highly recommendable.
